# Preclinical and first-in-human evidence of 4-hydroxybenzoic acid for mitochondrial COQ2 deficiency

**DOI:** 10.1093/brain/awaf334

**Published:** 2025-09-10

**Authors:** Felix Distelmaier, Julia Corral-Sarasa, Laura Jiménez-Sánchez, María Elena Díaz-Casado, Melanie Rohmann, Annette Seibt, Diran Herebian, Sander H J Smits, Juliane Münch, Ertan Mayatepek, Jörg Breitkreutz, Ralf A Husain, Sergio López-Herrador, Pilar González-García, Luis C López

**Affiliations:** Department of General Pediatrics, Neonatology and Pediatric Cardiology, University Children’s Hospital, Heinrich-Heine-University Düsseldorf, Düsseldorf 40225, Germany; MetabERN: European Reference Network for Rare Hereditary Metabolic Disorders, Health Care Provider Düsseldorf, Düsseldorf 40225, Germany; West German Center for Child and Adolescent Health, Partner Site Düsseldorf, Düsseldorf 40225, Germany; Departamento de Fisiología, Facultad de Medicina, Universidad de Granada, Granada 18016, Spain; Instituto de Investigación Biosanitaria ibs.Granada, Granada 18016, Spain; Departamento de Fisiología, Facultad de Medicina, Universidad de Granada, Granada 18016, Spain; Instituto de Investigación Biosanitaria ibs.Granada, Granada 18016, Spain; Departamento de Fisiología, Facultad de Medicina, Universidad de Granada, Granada 18016, Spain; Instituto de Investigación Biosanitaria ibs.Granada, Granada 18016, Spain; Department of Pediatrics, Pediatric Nephrology, Jena University Hospital, Jena 07747, Germany; Department of General Pediatrics, Neonatology and Pediatric Cardiology, University Children’s Hospital, Heinrich-Heine-University Düsseldorf, Düsseldorf 40225, Germany; West German Center for Child and Adolescent Health, Partner Site Düsseldorf, Düsseldorf 40225, Germany; Department of General Pediatrics, Neonatology and Pediatric Cardiology, University Children’s Hospital, Heinrich-Heine-University Düsseldorf, Düsseldorf 40225, Germany; West German Center for Child and Adolescent Health, Partner Site Düsseldorf, Düsseldorf 40225, Germany; Institute of Biochemistry and Center for Structural Studies, Heinrich-Heine-University, Düsseldorf 40225, Germany; Department of General Pediatrics, Neonatology and Pediatric Cardiology, University Children’s Hospital, Heinrich-Heine-University Düsseldorf, Düsseldorf 40225, Germany; West German Center for Child and Adolescent Health, Partner Site Düsseldorf, Düsseldorf 40225, Germany; Department of General Pediatrics, Neonatology and Pediatric Cardiology, University Children’s Hospital, Heinrich-Heine-University Düsseldorf, Düsseldorf 40225, Germany; MetabERN: European Reference Network for Rare Hereditary Metabolic Disorders, Health Care Provider Düsseldorf, Düsseldorf 40225, Germany; West German Center for Child and Adolescent Health, Partner Site Düsseldorf, Düsseldorf 40225, Germany; Institute of Pharmaceutics and Biopharmaceutics, Heinrich Heine University, Düsseldorf 40225, Germany; Center for Inborn Metabolic Disorders, Department of Neuropediatrics, Jena University Hospital, Jena 07747, Germany; Center for Rare Diseases, Jena University Hospital, Jena 07747, Germany; Departamento de Fisiología, Facultad de Medicina, Universidad de Granada, Granada 18016, Spain; Instituto de Investigación Biosanitaria ibs.Granada, Granada 18016, Spain; Departamento de Fisiología, Facultad de Medicina, Universidad de Granada, Granada 18016, Spain; Instituto de Investigación Biosanitaria ibs.Granada, Granada 18016, Spain; Departamento de Fisiología, Facultad de Medicina, Universidad de Granada, Granada 18016, Spain; Instituto de Investigación Biosanitaria ibs.Granada, Granada 18016, Spain; Centro de Investigación Biomédica en Red Fragilidad y Envejecimiento Saludable (CIBERFES), Granada 18016, Spain; Instituto de Biotecnología, Centro de Investigación Biomédica, Universidad de Granada, Granada 18016, Spain

**Keywords:** mitochondrial diseases, coenzyme Q deficiency, COQ2, 4-hydroxybenzoic acid, pharmacological therapy, therapeutic trial

## Abstract

Primary coenzyme Q (CoQ) deficiency is a mitochondrial disorder with variable clinical presentation and limited response to standard CoQ_10_ supplementation. Recent studies suggest that 4-hydroxybenzoic acid (4-HBA), a biosynthetic precursor of CoQ, may serve as a substrate enhancement treatment in cases caused by pathogenic variants in *COQ2*, a gene encoding a key enzyme in CoQ biosynthesis. However, it remains unclear whether 4-HBA is required throughout life to maintain health, whether it offers advantages over CoQ_10_ treatment, and whether these findings are translatable to humans.

Here, we demonstrate that lifelong 4-HBA supplementation in a murine model carrying the pathogenic *Coq2^A252V^* variant is well tolerated and prevents the onset of mitochondrial encephalopathy. In contrast, withdrawal of 4-HBA leads to progressive neurological decline. Notably, while conventional CoQ_10_ supplementation transiently ameliorated cardiac dysfunction, it failed to prevent fatal neurological deterioration.

Guided by these preclinical findings, we initiated a first-in-human individual therapeutic trial with 4-HBA in a 3-year-old boy with genetically confirmed primary CoQ_10_ deficiency due to compound heterozygous pathogenic *COQ2* variants. The patient presented with a Leigh-like syndrome characterized by bilateral brain lesions, developmental delay, muscular hypotonia, failure to thrive, lactic acidosis and steroid-resistant nephrotic syndrome. Despite high-dose oral CoQ_10_ supplementation, clinical response had been minimal. Prior to clinical application, patient-derived fibroblasts were treated *in vitro* with 4-HBA, resulting in a marked increase in endogenous CoQ_10_ levels.

Following the initiation of oral 4-HBA treatment, the patient experienced rapid and sustained remission of proteinuria, improved renal hyperfiltration and a gradual increase in serum CoQ_10_ concentrations. No adverse effects were observed during a 6-month follow-up. Clinically, the patient showed notable improvements in motor skills, language acquisition, cognitive alertness and overall development, accompanied by significant gains in growth and nutritional status. Clinical recovery was also reflected by improved scores on the Newcastle Paediatric Mitochondrial Disease Scale. These findings support 4-HBA as a promising targeted metabolic treatment for *COQ2*-related CoQ deficiency and highlight the need for further clinical investigation.

## Teaser

Distelmaier *et al.* show that supplementation with 4-hydroxybenzoic acid (4-HBA), a precursor of coenzyme Q, prevents the onset of mitochondrial encephalopathy in mice with *Coq2*-related CoQ deficiency, and produces striking clinical improvement in a child who had not responded to standard CoQ_10_ therapy.

## Introduction

Coenzyme Q (CoQ) is a key component of the mitochondrial respiratory chain and is essential for cellular energy production and metabolic homeostasis.^1-3^ Its endogenous biosynthesis requires a multistep process involving enzymes and regulatory proteins localized in both the cytosol and mitochondria.^2,3^ Pathogenic variants in genes encoding these proteins cause primary CoQ deficiency,^4^ a group of mitochondrial disorders with heterogeneous clinical presentation. Disease onset can range from the neonatal period, with lactic acidosis, encephalopathy and multiorgan failure, to adulthood, with slowly progressive neurological decline.^4,5^

Among these disorders, *COQ2*-related CoQ deficiency is particularly severe. *COQ2* encodes a polyprenyltransferase that catalyses the condensation of the benzoquinone ring (head group) with the polyisoprenoid side chain (tail group), a key step in CoQ biosynthesis (Supplementary Fig. 1). Pathogenic *COQ2* variants result in three major phenotypes,^4^ likely depending on residual enzyme activity: (i) a neonatal-onset form with encephalopathy, renal dysfunction and early death; (ii) an intermediate childhood-onset phenotype characterized by developmental delay, ataxia, muscular hypotonia, and steroid-resistant nephrotic syndrome progressing to neurodegeneration and kidney failure; and (iii) a late-onset, adult form presenting as a subtype of multiple system atrophy, predominantly reported in the Asian population.^6^

High-dose oral CoQ_10_ is the standard treatment for primary CoQ deficiency. However, clinical outcomes are often suboptimal.^7^ Several factors contribute to this poor efficacy, including low tissue, intracellular and intramitochondrial distribution, as well as its limited impact on CoQ_9_, another relevant CoQ form in humans.^7,8^ This limitation is particularly critical in patients with neurological involvement, as CoQ_10_ has limited ability to cross the blood–brain barrier and reach neuronal tissue, and the proportion of CoQ_9_ is higher in the brain than in other tissues, although its functional role remains unknown.^7-9^ In cases of pathogenic variants in *COQ2*, CoQ_10_ supplementation may provide transient renal improvement, particularly in nephrotic syndrome.^10^ Nonetheless, these effects are typically short-lived,^11^ and neurological symptoms generally remain unresponsive.^7^

To address this therapeutic gap, strategies aimed at reactivating endogenous CoQ biosynthesis through gene-specific treatment approaches are being explored. Two promising approaches are metabolic bypass or substrate enhancement treatments using small-molecule precursors of the CoQ biosynthetic pathway.^12^ These compounds offer superior physicochemical properties and may overcome the limitations of CoQ_10_. We previously demonstrated that supplementation with 4-hydroxybenzoic acid (4-HBA), the natural substrate of COQ2, restored endogenous CoQ_10_ biosynthesis in fibroblasts from patients carrying pathogenic *COQ2* variants.^13,14^  *In silico* analyses further suggested that COQ2 possesses a dedicated substrate channel guiding 4-HBA to its catalytic site. Mapping disease-associated pathogenic variants onto the model revealed that many of them affect this transport pathway, potentially disrupting enzymatic activity.^13^ These findings led us to hypothesize that increasing 4-HBA availability could compensate for defective substrate delivery and stimulate residual COQ2 activity.

To test this hypothesis *in vivo*, we recently developed a knock-in mouse model carrying the pathogenic *Coq2^A252V^* variant. Homozygous animals displayed perinatal lethality with multisystemic disease, including cardiomyopathy, oedema and neurodevelopmental delay, closely recapitulating the human neonatal phenotype.^14^ Treatment with 4-HBA led to an increase in CoQ_9_ and CoQ_10_ levels, resulting in improved function of the mitochondrial respiratory chain. Consequently, 4-HBA rescued perinatal lethality and multisystemic disease in *Coq2^A252V^* mice.^14,15^ However, whether 4-HBA is required throughout life to maintain health, whether it is superior to CoQ_10_ treatment, and whether these findings are translatable to humans remain unknown.

Here, we show that continuous 4-HBA treatment is essential to prevent neurological deterioration in *Coq2^A252V^* mice. Withdrawal of treatment triggered progressive mitochondrial encephalopathy. In contrast, CoQ_10_ supplementation rescued cardiac symptoms but failed to prevent fatal neurological decline. Given the results in the mouse model, we initiated a first-in-human individual therapeutic trial with 4-HBA in a child carrying two compound heterozygous variants in *COQ2*. We demonstrate that oral 4-HBA is safe and clinically effective, highlighting its potential as a targeted metabolic therapy in primary CoQ deficiency.

## Material and methods

### Ethics statement

Procedures in mice were approved by the Institutional Animal Care and Use Committee of the University of Granada (protocol number 30/06/2022/097) and were conducted in compliance with the European Convention for the Protection of Vertebrate Animals Used for Experimental and Other Scientific Purposes (CETS #123) and Spanish legislation (R.D. 53/2013).

The compassionate-use protocol in the patient was thoroughly reviewed with institutional experts, including the hospital pharmacy and the Institute of Pharmaceutics and Biopharmaceutics. Ethical approval was obtained from the local ethics committee at the University Hospital of Düsseldorf. The study adheres to good clinical practice and follows the Declaration of Helsinki. Written informed consent for the use of photographs and videos of the minor was obtained from the parents.

### Animals and treatments

This study used *Coq2^+/+^* (wild-type) and *Coq2^A252V^* mice, both with C57BL/6J genetic background. This mouse mutation mirrors the pathogenic p.Ala302Val (A302V) variant in the human *COQ2* gene, previously reported in two cases. Both individuals were born prematurely by cesarean section due to signs of fetal distress. Shortly after birth, they developed severe clinical symptoms, including feeding intolerance, widespread oedema, seizures and apnoea, ultimately resulting in death before reaching 6 months of age.^15^ The *Coq2^A252V^* model has been previously generated and characterized.^14^ Owing to the perinatal lethality of the *Coq2^A252V^* genotype, only animals rescued by treatment with 4-HBA or CoQ_10_ were included.^14^

4-HBA (Merck Life Science S.L.U) and CoQ_10_ (Fagron NV) were orally administered through incorporation into chow at a concentration of 0.33% (w/w). The efficacy of 4-HBA at this dose was previously demonstrated in *Coq2^A252V^* mice.^14^

Treatment was started in females with the mating of *Coq2^+/A252V^* mice, and analyses were performed at 21 days of age. Comparisons were made with untreated *Coq2^+/+^* mice, as *Coq2^A252V^* mice do not survive the perinatal period.

To evaluate the effect of 4-HBA treatment discontinuation, *Coq2^A252V^* mice were treated with 4-HBA for 90 days, until they reached adulthood. At 90 days of age, the 4-HBA treatment was discontinued. Measurements were performed at 160 days of age (corresponding to 70 days without 4-HBA treatment), since differences between groups began to appear at this time point, and at 300 days of age (corresponding to 210 days without 4-HBA treatment), because this is the moment at which the mice in this experimental group start to die. Comparisons were made with the group that received continuous 4-HBA treatment (*Coq2^A252V^* + 4-HBA).

Mice were housed under specific pathogen-free (SPF) conditions in the Animal Facility of the University of Granada, with a 12-h light/dark cycle (lights on at 7:00 a.m. and off at 7:00 p.m.) and *ad libitum* access to water and a standardized rodent chow diet (SAFE® 150), which provides 21% of energy from protein, 12.6% from lipids and 66.4% from nitrogen-free extracts. In the analytical experiments, we used an equal number of male and female mice.

### Mouse motor function tests

For the 4-HBA discontinuation study, body weight gains were recorded every 2 weeks until a maximum of 40 weeks, as after this period the mice began to die.

In the comparative study of CoQ_10_ versus 4-HBA treatments, body weights were recorded at 21 days of age. Motor tests were also conducted at 21 days of age, as most of the CoQ_10_-treated mice died before reaching 1 month of age.

The beam balance test involved measuring the time taken for the mouse to cross a 1-m-long bar with a 1-cm-wide surface, held at a height of 50 cm. A total maximum time of 60 s was allowed to complete the task, and if the mouse did not finish within this time, the maximum time was assigned.

### Quantification of CoQ_9_ and CoQ_10_ levels in animal tissues

Tissue lipid extraction was carried out by mixing the tissue homogenate with 1-propanol, followed by vortexing for 30 s and centrifugation at 11 300*g* for 5 min. The resulting supernatant was injected into the high-performance liquid chromatography (HPLC) system using a reverse-phase C18 3.5 μm, 4.6 × 150 mm column (Waters) and a mobile phase consisting of methanol, ethanol, 2-propanol, acetic acid (500:500:15:15) and 50 mM sodium acetate at a flow rate of 0.9 ml/min.^16^ CoQ_9_ and CoQ_10_ levels were quantified in the extract using reversed-phase HPLC coupled to electrochemical detection, as previously described.^16^ Subsequently, a standard curve of CoQ_9_ and CoQ_10_ was used for quantification, and results were normalized by milligrams of protein, which were determined in tissue homogenates using the Bradford assay. Results were expressed as nanograms of CoQ per milligram of protein.^16^

### Assessment of mitochondrial respiration

Mitochondria were isolated from the kidneys and brain as previously described.^17^ The isolated mitochondria were diluted in cold 1× MAS buffer and plated at a concentration of 1.5 mg/well for kidney and 3.5 mg/well for brain.^17^ A 50 µl aliquot of mitochondrial suspension was added to each well (excluding background correction wells) while the plate was kept on ice, then centrifuged at 2000*g* for 10 min at 4°C. Following centrifugation, 450 µl of 1× MAS buffer, supplemented with 10 mM succinate, 2 mM malate, 2 mM glutamate and 10 mM pyruvate, were added to each well. Injections were performed as follows: port A, 50 µl of 40 mM ADP (final concentration: 4 mM); port B, 55 µl of 30 mg/ml oligomycin (final concentration: 3 mg/ml); port C, 60 µl of 40 mM FCCP (final concentration: 4 mM); and port D, 65 µl of 40 mM antimycin A (final concentration: 4 mM). Mitochondrial respiration was assessed using an XFe^24^ Extracellular Flux Analyzer (Seahorse Bioscience), and all data were expressed as oxygen consumption rate (OCR, pmol/min).^17^

### Histology and immunofluorescence

For histology experiments, tissues were fixed in 4% paraformaldehyde for 24 h, processed and embedded in paraffin. Multiple sections (4 µm thick) were deparaffinized with xylene and stained with haematoxylin and eosin.^17^

To evaluate renal morphology, we performed Periodic acid-Schiff (PAS) staining, which highlights the glomerular basement membrane and glycogen in the renal tubules. The kidneys were fixed in 4% paraformaldehyde for 24 h, processed and embedded in paraffin. Multiple sections (4-µm thick) were deparaffinized and incubated in 1% periodic acid. Then, staining was performed using Schiff’s reagent and haematoxylin. Sections were examined under ×10 to ×40 magnification with a Nikon Eclipse Ni-U microscope (Werfen), and the images were scanned under identical lighting conditions using NIS-Elements BR software (Werfen).

To identify neurons (NeuN, 1:300, Merck Millipore), myelin (anti-MBP, 1:500, Abcam), astrocytes (GFAP, 1:500, Sigma Aldrich) and microglial activation (Iba1, 1:500, Wako), immunohistochemistry was performed.^17^ Briefly, after deparaffinization, sections were boiled using 0.1 M sodium citrate buffer (pH 6) at 90°C for 40 min. After several washes in PBS (0.1 M, 0.02% Triton X-100), sections were incubated with the primary antibody at 4°C overnight. Secondary antibodies conjugated with Alexa Fluor 488 or 594 were then applied at 37°C for 2 h. Finally, slides were mounted with ProLong™ Gold Antifade Mountant with DAPI (Invitrogen).^17^ Sections were examined at ×40 to ×400 magnification with a Nikon Eclipse Ni-U microscope (Werfen), and images were scanned under identical light conditions using NIS-Elements BR software (Werfen).

### Cell culture

Human primary skin fibroblasts were cultured in Dulbecco’s modified Eagle medium (Life Technologies) supplemented with 10% fetal bovine serum (Life Technologies) and 1% penicillin/streptomycin (Life Technologies) at 37°C in a humidified atmosphere of 5% CO_2_. Treatment was done with 100 µM 4-HBA as described previously.^13^

### UPLC-ESI-MS/MS analysis of fibroblasts and serum samples

Ultra-high performance liquid chromatography-electrospray ionization tandem mass spectrometry (UPLC-ESI-MS/MS) analysis of fibroblast and serum samples was performed as described previously, using an Acquity UPLC-I Class (Waters) coupled to a Waters Xevo TQ-XS tandem mass spectrometer (Waters), which was equipped with an ESI source operating in the positive ion mode.^13^ Serum CoQ_10_ levels were consistently measured under standardized conditions (morning samples, 3 h post-prandial).

### *In silico* analysis

The apo structure of the archaeal homologue of COQ2 from *Aeropyrum pernix* was used as a template to build a model of human COQ2 using the modelling server Phyre2, for the wild-type as well as variant sequences.^13^ The resulting structural model was manually inspected using the program COOT.^13^ We compared the obtained model with the model calculated with Alphafold (Uniprot Q96H96), which became available near the end of this study, using the Pymol align tool.^18^ The 4-HBA binding site was determined using the program AUTODOCK.^13^ After inspection of protein ligand interactions, putative binding sites were visualized using the program Pymol (http://www.pymol.org).

### First-in-human treatment with 4-HBA

To date, 4-HBA has not been used as a drug or dietary supplement. However, 4-HBA derivatives are commonly used as additives in the food and cosmetic industries (e-numbers E214 and E215). Notably, 4-HBA holds GRAS (‘Generally Recognized As Safe’) status by the US Food and Drug Administration (FDA).

As an organic acid, 4-HBA carries a potential risk of irritation to eyes, skin and mucous membranes. However, its acid dissociation constant (pKa = 4.61) is comparable to that of acetic acid (pKa = 4.76), and less acidic than acetylsalicylic acid (pKa = 3.48), a widely used pharmaceutical. Therefore, oral administration of 4-HBA is considered safe when appropriately diluted in milk or water.

Beyond extensive toxicological studies in rodents, 4-HBA has also been administered orally to human subjects in research settings. In these studies, individuals received up to 5 g every 6 h for 24 h, or a total of 26 g over 28 h, diluted in drinking water. These regimens were well tolerated, with no adverse effects reported (https://gestis.dguv.de/data?name=492602&lang=en).

Based on the available preclinical data, including patient-derived fibroblasts and a murine model, and the safety data outlined above, we initiated an individual therapeutic trial with 4-HBA in the before mentioned patient. 4-HBA (≥99.9% purity) was obtained from BLD Pharmatech GmbH and further tested for potential contaminants by Taros Chemicals. Encapsulation of 4-HBA at specific dosages was performed by the hospital pharmacy. The human equivalent dose was calculated by correcting the mouse dose with the body surface area.^19^

### Statistical analysis

No statistical method was applied to determine sample size. Statistical analyses were conducted using Prism 10 scientific software. Data are presented as mean ± standard deviation. In Fig. 4D–K, each point represents a biological replicate. A one-way ANOVA with a Tukey’s *post hoc* test was used to compare the differences between the three experimental groups. Studies with two experimental groups were evaluated using unpaired Student’s *t*-test. A *P*-value of <0.05 was considered to be statistically significant.

## Results

### Discontinuing 4-HBA treatment in adult *Coq2^A252V^* mice leads to progressive mitochondrial encephalopathy

Recently, we demonstrated that 4-HBA supplementation, administered orally through the pregnant female, increases the endogenous CoQ levels and rescues the perinatal lethality in *Coq2^A252V^* mice.^14^ These data demonstrated that COQ2 is critical during embryonic development and that 4-HBA could overcome the limitation of having a dysfunctional COQ2 protein. Thus, born mutant mice are indistinguishable from wild-type mice if the animals are daily supplemented with 4-HBA.^14^ To evaluate whether COQ2 is also required in adult life and if 4-HBA supplementation must be continuously administered in individuals with pathogenic variants in *Coq2*, we discontinued 4-HBA therapy in *Coq2^A252V^* mice. Therefore, *Coq2^A252V^* mice were treated with 4-HBA for 90 days until they reached adulthood. At that time point, the treatment was discontinued.

**Figure 1:**
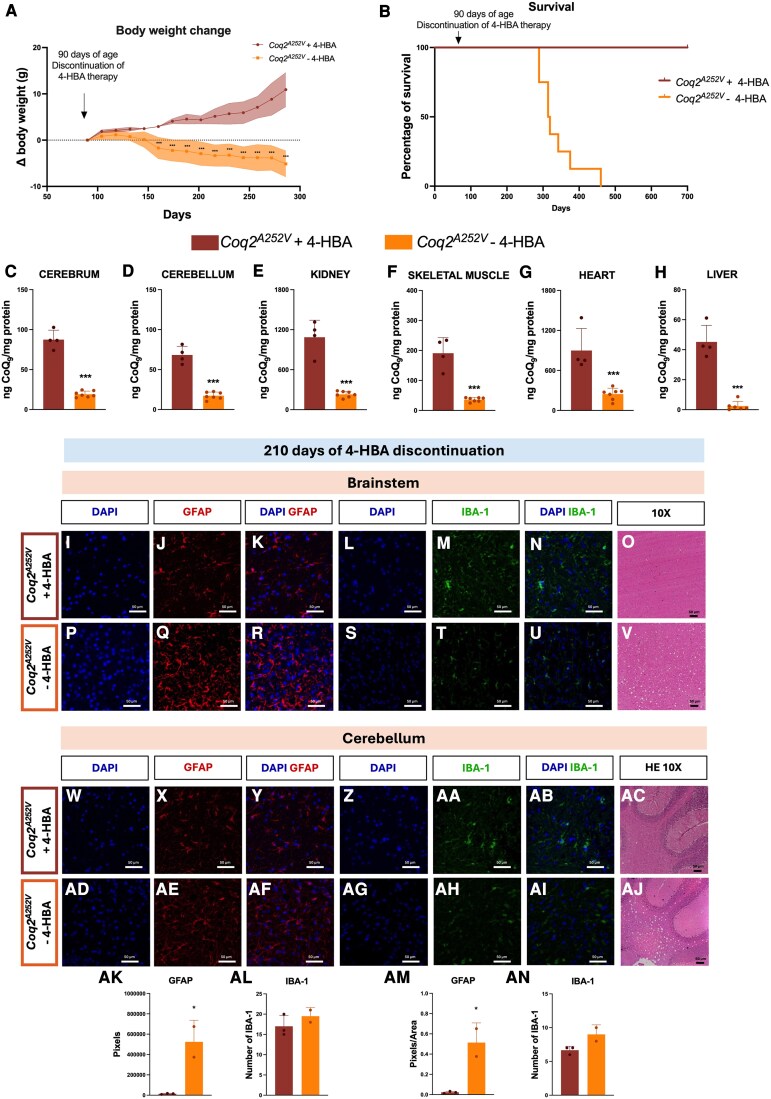
**Phenotypic and metabolic impact of 4-HBA discontinuation after 90 days of treatment in *Coq2^A252V^* mice.** (**A**) Body weight change after discontinuation of 4-HBA treatment (*n* = 6–8 in each experimental group). (**B**) Survival curve after discontinuation of 4-HBA treatment, comparing the experimental groups: *Coq2^A252V^* treated continuously with 4-HBA; and *Coq2^A252V^* treated with 4-HBA for the first 90 days only (*n* = 6–8 in each experimental group). (**C–H**) CoQ_9_ levels in cerebrum (**C**), cerebellum (**D**), kidney (**E**), skeletal muscle (**F**), heart (**G**) and liver (**H**) at Day 160 of age, after 70 days with or without 4-HBA treatment (*n* = 4–7 in each experimental group). (**I–K**) GFAP stain and (**L–N**) IBA-1 stain in the brainstem of *Coq2^A252V^* mice treated with 4-HBA at 210 days post-withdrawal (*n* = 3–5 in each experimental group). (**O**) Haematoxylin-eosin stain in the brainstem of *Coq2^A252V^* mice treated with 4-HBA at 210 days post-withdrawal (*n* = 3–5 in each experimental group). (**P–R**) GFAP stain and (**S–U**) IBA-1 stain in the brainstem of *Coq2^A252V^* mice after 4-HBA discontinuation at 210 days post-withdrawal (*n* = 3–5 in each experimental group). (**V**) Haematoxylin-eosin stain in the brainstem of *Coq2^A252V^* mice after 4-HBA discontinuation at 210 days post-withdrawal (*n* = 3–5 in each experimental group). (**W–Y**) GFAP stain and (**Z–AB**) IBA-1 stain in the cerebellum of *Coq2^A252V^* mice treated with 4-HBA at 210 days post-withdrawal (*n* = 3–5 in each experimental group). (**AC**) Haematoxylin-eosin stain in the cerebellum of *Coq2^A252V^* mice treated with 4-HBA at 210 days post-withdrawal (*n* = 3–5 in each experimental group). (**AD–AF**) GFAP stain and (**AG–AI**) IBA-1 stain in the cerebellum of *Coq2^A252V^* mice after 4-HBA discontinuation at 210 days post-withdrawal (*n* = 3–5 in each experimental group). (**AJ**) Haematoxylin-eosin stain in the cerebellum of *Coq2^A252V^* mice after 4-HBA discontinuation at 210 days post-withdrawal (*n* = 3–5 in each experimental group). (**AK**) Quantification in pixels of GFAP in brainstem of *Coq2^A252V^* mice treated with 4-HBA and after 4-HBA discontinuation at 210 days post-withdrawal (*n* = 2–3 in each experimental group). (**AL**) Quantification of IBA-1 in brainstem of *Coq2^A252V^* mice treated with 4-HBA and after 4-HBA discontinuation at 210 days post-withdrawal (*n* = 2–3 in each experimental group). (**AM**) Quantification in pixels of GFAP in cerebellum of *Coq2^A252V^* mice treated with 4-HBA and after 4-HBA discontinuation at 210 days post-withdrawal (*n* = 2–3 in each experimental group). (**AN**) Quantification of IBA-1 in cerebellum of *Coq2^A252V^* mice treated with 4-HBA and after 4-HBA discontinuation at 210 days post-withdrawal (*n* = 2–3 in each experimental group). Scale bars = 50 µm (**I–AJ**). *Coq2^A252V^* mice were treated with 4-HBA for 90 days. The 4-HBA was given in the chow at a concentration of 0.33% (w/w). At that point, the treatment was discontinued and replaced with standard animal facility chow in the *Coq2^A252V^* without 4-HBA group. Data are presented as mean ± standard deviation. ***P < 0.001 versus *Coq2^A252V^* treated with 4-HBA. 4-HBA = 4-hydroxybenzoic acid.

Progressive weight loss was observed in *Coq2^A252V^* mice after 4-HBA discontinuation. By 160 days of age (70 days without treatment), progressive weight loss in these mice became evident, with differences becoming more pronounced over time (Fig. 1A). This weight loss was associated with reduced survival. Around 300 days of age, equivalent to 210 days without treatment, the survival rate of mice in which 4-HBA treatment was discontinued began to decline, reaching a maximum lifespan of 460 days. Comparatively, all *Coq2^A252V^* mice treated with 4-HBA remained alive at the same age (Fig. 1B), and we currently have both male and female *Coq2^A252V^* mice that have reached 700 days of age. Interestingly, at the final stage, *Coq2^A252V^* mice in which 4-HBA therapy was discontinued exhibited abnormal forelimb and hindlimb movements (Supplementary Video 1), suggestive of mitochondrial encephalopathy with possible involvement of the pyramidal tracts.

Given that the fatal phenotype may be attributed to impaired CoQ metabolism, we assessed CoQ levels in *Coq2^A252V^* mice at 160 days of age, 70 days after the discontinuation of 4-HBA therapy, the time point at which weight loss began. Our analysis revealed that discontinuation of 4-HBA therapy in *Coq2^A252V^* mice lead to reduced levels of CoQ_9_, CoQ_10_ and the ratio CoQ_9_/CoQ_10_ in the cerebrum, cerebellum, kidney, skeletal muscle, heart and liver compared to 4-HBA-treated *Coq2^A252V^* mice (Fig. 1C–H and Supplementary Fig. 2A–L).

To characterize the encephalopathic phenotype in *Coq2^A252V^* mice after 4-HBA withdrawal (Supplementary Video 1), we analysed the brain at 210 days following treatment discontinuation, since at this stage, the onset of mortality was observed among the animals. We observed strong astrocyte activation in the brainstem of animals in which 4-HBA treatment was discontinued (Fig. 1P–R and AK), compared to mice that continued receiving the treatment (Fig. 1I–K and AK). However, no microglial activation was detected in this region of the same animals (Fig. 1L–N, S–U and AL). In the cerebellum, astrocyte activation was also observed, although it was less pronounced compared to that seen in the brainstem (Fig. 1W–Y, AD–AF and AM). No evidence of microglial activation was found in this region (Fig. 1Z–AB, AG–AI and AN). Notably, brainstem and cerebellar vacuolation was detected in mice following 4-HBA withdrawal, a finding consistent with the spongiosis observed in other mouse models of mitochondrial encephalopathy,^20,21^ which may account for the encephalopathic phenotype observed in this model (Fig. 1O–V and AC–AJ).

To understand how 4-HBA withdrawal affects the brain over time, we also evaluated the animals an earlier stage, at 70 days, when CoQ levels were already low, and weight loss was starting to become evident. At this time, we observed disorganization of myelin in the cortex of untreated mice (Supplementary Fig. 2M–O, S–U and Y), although no changes in the number of neurons were found in this area (Supplementary Fig. 2P–R, V–X and Z). In contrast, astrocyte activation was higher in the brainstem of mice who did not receive 4-HBA treatment (Supplementary Fig. 2AA–AG), but this was not observed in the cerebellum (Supplementary Fig. 2AH–AJ, AN–AP and AT). Regarding microglia, no significant differences were observed in this area (Supplementary Fig. 2AK–AM, AQ–AS and AU).

These results suggest that neurological damage develops progressively, becoming more evident after prolonged treatment withdrawal. Altogether, our data indicate that chronic administration of 4-HBA is necessary for applications in a potential translation of this treatment in patients.

### 4-HBA treatment has superior therapeutic outcomes than standard CoQ10 treatment

**Figure 2:**
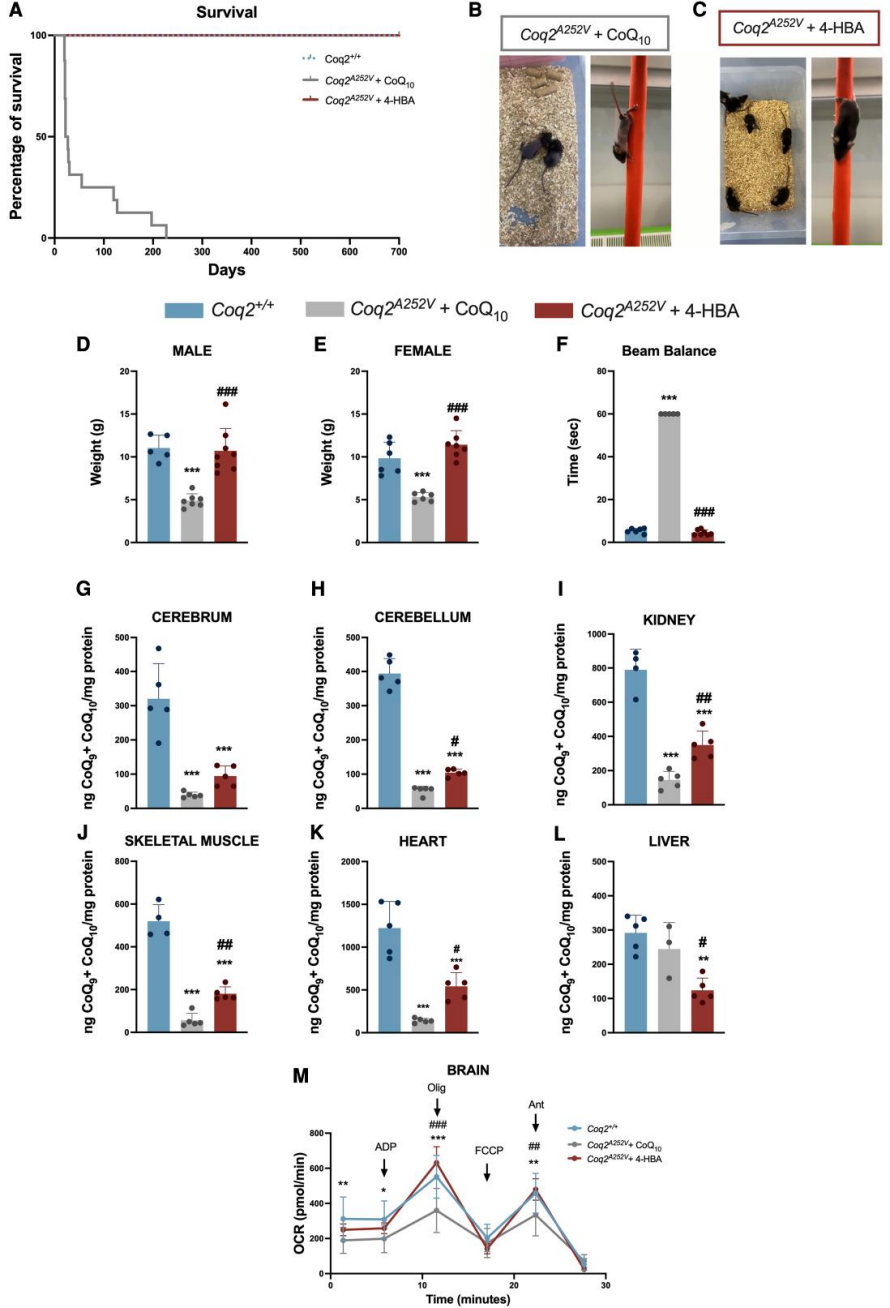
**Comparison of the effect of CoQ_10_ versus 4-HBA treatment on survival, mitochondrial function in *Coq2^A252V^* mice.** (**A**) Survival curve from the experimental groups of *Coq2^+/+^*, *Coq2^A252V^* mice treated with CoQ_10_ and *Coq2^A252V^* mice treated with 4-HBA (*n* = 16–20 in each experimental group). (**B** and **C**) Representative photos of a pole test conducted by *Coq2^A252V^* mice treated with CoQ10 (**B**) and *Coq2^A252V^* mice treated with 4-HBA (**C**), at 21 days of age. (**D** and **E**) Body weight of male (**D**) and female (**E**) mice at 21 days of age (*n* = 5–8 in each experimental group). (**F**) Beam balance test in mice at 21 days of age (*n* = 5–7 in each experimental group). (**G–L**) Levels of total CoQ (CoQ_9_ + CoQ_10_) in cerebrum (**G**), cerebellum (**H**), kidney (**I**), skeletal muscle (**J**), heart (**K**) and liver (**L**) of mice at 21 days of age (*n* = 3–5 in each experimental group). (**M**) Mitochondrial oxygen consumption rate (represented as State 3o, in the presence of ADP and substrates) in brain (*n* = 3 in each experimental group) of mice at 21 days of age. Ant = antimycin; Olig = oligomycin. CoQ_10_ and 4-HBA were given to the mice in the chow at a concentration of 0.33% (w/w). Data are presented as mean ± standard deviation. *P < 0.05 versus *Coq2^+/+^*, **P < 0.01 versus *Coq2^+/+^*, ***P < 0.001 versus *Coq2^+/+^*, ^#^*P* < 0.05 versus *Coq2^A252V^* treated with CoQ_10_, ^##^*P* < 0.01 versus *Coq2^A252V^* treated with CoQ_10_, ^###^*P* < 0.001 versus *Coq2^A252V^* treated with CoQ_10_. 4-HBA = 4-hydroxybenzoic acid.

Since the standard treatment for patients with primary CoQ deficiency is oral CoQ_10_, we decided to compare CoQ_10_ and 4-HBA treatments at the same dose in *Coq2^A252V^* mice. The survival study revealed that both 4-HBA and CoQ_10_ treatments successfully rescued the perinatal lethality observed in *Coq2^A252V^* mice. However, a rapid decline in survival was observed in CoQ_10_-treated mice, in comparison to those treated with 4-HBA. The survival rate of CoQ_10_-treated mice dropped to 30% at 30 days of age, with a maximum lifespan of 240 days, which was only reached by 5% of the CoQ_10_-treated mice. In contrast, at 240 days of age, 100% of the mice treated with 4-HBA and *Coq2^+/+^* mice were still alive (Fig. 2A). So far, we have 4-HBA-treated mutant mice that have reached 700 days of age, and they appear completely normal, both males and females.

At 21 days of age, phenotypic differences between treatments were observed (Fig. 2B and C and Supplementary Video 2). In both males and females, the body weight of CoQ_10_-treated mice was reduced by half compared to *Coq2^+/+^* mice and *Coq2^A252V^* mice treated with 4-HBA (Fig. 2D and E). Around this age, CoQ_10_-treated mice lose their body hair, but it grows back during the next hair-growth cycle if the animals are still alive, a phenomenon that has also been observed in other mouse models of mitochondrial diseases.^20,22^ Additionally, *Coq2^A252V^* mice treated with CoQ_10_ developed severe motor impairments, particularly affecting hind limb function. Owing to these deficits, the pole test could not be performed properly, as the animals were unable to grasp the vertical pole and frequently fell, posing a risk of injury (Supplementary Video 3). Moreover, these mice were unable to complete other motor assessments, such as the beam balance test. Consequently, they were assigned the maximum allowed time, 60 s (Fig. 2F). In addition, these mice exhibited tremors and impaired balance, in contrast to those treated with 4-HBA, which displayed normal behaviour (Supplementary Video 4).

The decreased survival and impaired motor function of *Coq2^A252V^* mice treated with CoQ_10_ appear to be related to the treatment’s inability to increase CoQ levels. In fact, CoQ_10_-treated mice show lower levels of total CoQ (CoQ_9_ + CoQ_10_) in the cerebrum (Fig. 2G), cerebellum (Fig. 2H), kidney (Fig. 2I), skeletal muscle (Fig. 2J) and heart (Fig. 2K) compared to the mice that received 4-HBA treatment. Only the liver showed an increase of total CoQ levels after CoQ_10_ supplementation (Fig. 2L). That change was due to an increase in CoQ_10_ levels (Supplementary Fig. 3L), with no changes observed in CoQ_9_ levels (Supplementary Fig. 3F). The impairment of CoQ synthesis affected both CoQ_9_ and CoQ_10_, since there was a decrease of both in the cerebrum (Supplementary Fig. 3A and G), cerebellum (Supplementary Fig. 3B and H), kidney (Supplementary Fig. 3C and I), skeletal muscle (Supplementary Fig. 3D and J) and heart (Supplementary Fig. 3E and K).

Decreased CoQ is reflected by mitochondrial respiration in the brain. The mitochondrial OCR was lower in the brain of CoQ_10_-treated *Coq2^A252V^* mice compared to 4-HBA-treated *Coq2^A252V^* mice or *Coq2^+/+^* mice. No differences in OCR were found between *Coq2^A252V^* mice treated with 4-HBA and *Coq2^+/+^* mice (Fig. 2M). In the kidney, however, OCR levels were normalized with both treatments (Supplementary Fig. 3M), suggesting that the CoQ levels produced in this tissue are high enough to support its bioenergetics requirements.

**Figure 3:**
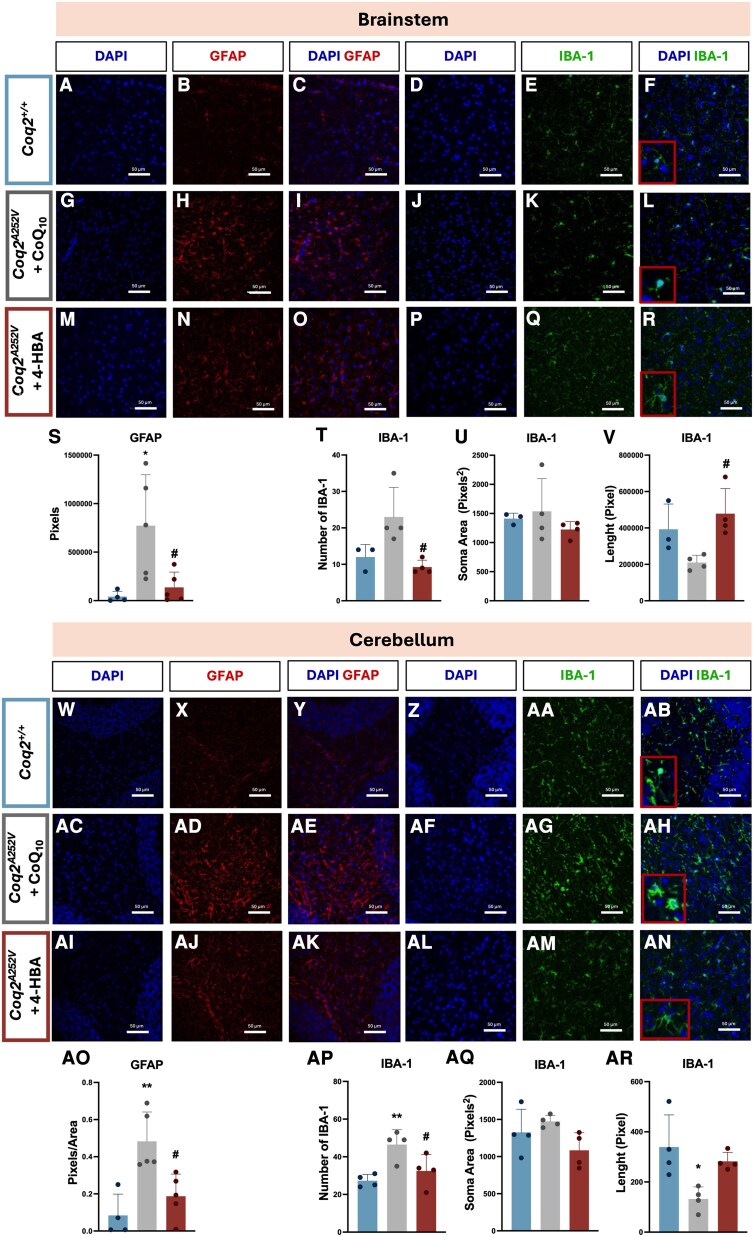
**Evaluation of CoQ_10_ and 4-HBA treatment outcomes on neuroinflammation in *Coq2^A252V^* mice.** (**A–C**) GFAP stain and (**D–F**) IBA-1 stain in the brainstem of *Coq2^+/+^* mice at 21 days of age (*n* = 4–5 in each experimental group). (**G–I**) GFAP stain and (**J–L**) IBA-1 stain in the brainstem of *Coq2^A252V^* mice treated with CoQ_10_, at 21 days of age (*n* = 4–5 in each experimental group). (**M–O**) GFAP stain and (**P–R**) IBA-1 stain in the brainstem of *Coq2^A252V^* mice treated with 4-HBA, at 21 days of age (*n* = 4–5 in each experimental group). (**S**) Quantification in pixels of GFAP in brainstem of *Coq2^+/+^*, *Coq2^A252V^* mice treated with CoQ_10_ and *Coq2^A252V^* mice treated with 4-HBA, at 21 days of age (*n* = 4–5 in each experimental group). (**T**) Quantification of IBA-1 in brainstem of *Coq2^+/+^*, *Coq2^A252V^* mice treated with CoQ_10_ and *Coq2^A252V^* mice treated with 4-HBA, at 21 days of age (*n* = 4 in each experimental group). (**U**) Quantification of the soma area of IBA-1 in pixels in brainstem of *Coq2^+/+^*, *Coq2^A252V^* mice treated with CoQ_10_ and *Coq2^A252V^* mice treated with 4-HBA, at 21 days of age (*n* = 4 in each experimental group). (**V**) Quantification of the length of IBA-1 in pixels in brainstem of *Coq2^+/+^*, *Coq2^A252V^* mice treated with CoQ_10_ and *Coq2^A252V^* mice treated with 4-HBA, at 21 days of age (*n* = 4 in each experimental group). (**W–Y**) GFAP stain and (**Z–AB**) IBA-1 stain in the cerebellum of *Coq2^+/+^* mice at 21 days of age (*n* = 4–5 in each experimental group). (**AC–AE**) GFAP stain and (**AF–AH**) IBA-1 stain in the cerebellum of *Coq2^A252V^* mice treated with CoQ_10_, at 21 days of age (*n* = 4–5 in each experimental group). (**AI–AK**) GFAP stain and (**AL–AN**) IBA-1 stain in the cerebellum of *Coq2^A252V^* mice treated with 4-HBA, at 21 days of age (*n* = 4–5 in each experimental group). (**AO**) Quantification in pixels of GFAP in cerebellum of *Coq2^+/+^*, *Coq2^A252V^* mice treated with CoQ_10_ and *Coq2^A252V^* mice treated with 4-HBA, at 21 days of age (*n* = 4–5 in each experimental group). (**AP**) Quantification of IBA-1 in cerebellum of *Coq2^+/+^*, *Coq2^A252V^* mice treated with CoQ_10_ and *Coq2^A252V^* mice treated with 4-HBA, at 21 days of age (*n* = 4 in each experimental group). (**AQ**) Quantification of the soma area of IBA-1 in pixels in cerebellum of *Coq2^+/+^*, *Coq2^A252V^* mice treated with CoQ_10_ and *Coq2^A252V^* mice treated with 4-HBA, at 21 days of age (*n* = 4 in each experimental group). (**AR**) Quantification of the length of IBA-1 in pixels in cerebellum of *Coq2^+/+^*, *Coq2^A252V^* mice treated with CoQ_10_ and *Coq2^A252V^* mice treated with 4-HBA, at 21 days of age (*n* = 4 in each experimental group). Scale bars = 50 µm (**A–R** and **W–AN**). CoQ_10_ and 4-HBA were given to the mice in the chow at a concentration of 0.33% (w/w). Data are presented as mean ± standard deviation. *P < 0.05 versus *Coq2^+/+^*, **P < 0.01 versus *Coq2^+/++^*, ^#^*P* < 0.05 versus *Coq2^A252V^* treated with CoQ_10_. 4-HBA = 4-hydroxybenzoic acid.

As the CoQ levels, mitochondrial respiration and phenotypic characterization suggested that the CNS in the CoQ_10_-treated mice was compromised, we morphologically evaluated the brain in different areas. Thus, in the brainstem, morphological changes were observed in the astrocytes of *Coq2^A252V^* mice treated with CoQ_10_, as they exhibited a scar phenotype, indicating signs of reactive astrogliosis (Fig. 3G–I and S). In contrast, *Coq2^A252V^* mice treated with 4-HBA displayed astrocytes with normal phenotype (Fig. 3M–O and S), similar to the *Coq2^+/+^* mice (Fig. 3A–C and S). In the brainstem, microglia from CoQ_10_-treated mice displayed an inflammatory M1 phenotype and were more numerous compared to those in *Coq2^+/+^* and 4-HBA-treated *Coq2^A252V^* mice. This phenotype was marked by reduced branching and an enlarged soma, indicative of reactive microglia (Fig. 3D–F, J–L, P–R and T–V). In the cerebellum, we observed hyperactivation of reactive astrocytes in the CoQ_10_-treated *Coq2^A252V^* mice (Fig. 3AC–AE and AO), similar to observations in the brainstem of the same animals, but not in 4-HBA-treated *Coq2^A252V^* mice or *Coq2^+/+^* mice (Fig. 3AI–AK, W–Y and AO). In this region, microglia were more activated and exhibited an inflammatory M1 phenotype in CoQ_10_-treated mice, in contrast to the 4-HBA-treated *Coq2^A252V^* mice and *Coq2^+/+^* mice (Fig. 3Z–AB, AF–AH, AL–AN and AP–AR). In the cortex, there was an increased number of microglia despite an apparent reduction in their activation state in CoQ_10_-treated mice, while no differences were observed in the number of neurons between the different groups (Supplementary Fig. 3N–S, V–AA, AD–AI and AL–AN). Overall, these data suggest that CoQ_10_-treated *Coq2^A252V^* mice exhibit neuroinflammation in the brainstem and cerebellum, in contrast to the protective effect of the 4-HBA therapy.

Additionally, since patients with pathogenic variants in *COQ2* typically develop steroid-resistant nephrotic syndrome,^23^ we examined kidney morphology in the experimental groups using PAS staining. In *Coq2^+/+^* mice (Supplementary Fig. 3T and U) and *Coq2^A252V^* mice treated with 4-HBA (Supplementary Fig. 3AJ and AK), renal tubules and glomeruli displayed normal morphology. However, in *Coq2^A252V^* mice treated with CoQ_10_ (Supplementary Fig. 3AB and AC) exhibited hypertrophic glomeruli, which may indicate an early sign of nephropathy.

Collectively, these data, summarized in Table 1, strongly suggest that patients with pathogenic variants in *COQ2* should preferentially be treated with 4-HBA rather than CoQ_10_.

**Table 1:** Summary of the most relevant results obtained in the three experimental groups of the *Coq2^A252V^* mouse model

Feature	*Coq2^A252V^* + 4-HBA	*Coq2^A252V^* + CoQ_10_	*Coq2^A252V^* − 4-HBA
Maximum survival	>700 days (mice still alive at study end point)	<240 days	<460 days
Body weight	Normal	50% reduction in body weight compared to WT	Progressive weight loss following 4-HBA withdrawal
Behavioural alterations	Normal behaviour	Severe motor impairment including hindlimb weakness, tremor, and postural instability	Abnormal limb movements suggestive of mitochondrial encephalopathy with pyramidal signs
CoQ levels in the brain	Partial restoration of CoQ_9_ and CoQ_10_ levels in cerebrum and cerebellum	CoQ_9_ and CoQ_10_ levels remain low in brain and cerebellum. Associated with impaired mitochondrial respiration	Marked decrease in CoQ_9_ and CoQ_10_ levels in the cerebrum and cerebellum
CoQ levels in other tissues	Partial restoration of CoQ_9_ and CoQ_10_ levels in kidney, skeletal muscle, heart and liver	CoQ_9_ and CoQ_10_ levels do not increase in kidney, skeletal muscle and heart. Only CoQ_10_ levels are normalized in the liver	Marked decrease in CoQ_9_ and CoQ_10_ levels in the kidney, skeletal muscle, heart and liver
Histopathological signs in the brain	No alterations detected	Astrogliosis and microgliosis, predominantly in the brainstem and cerebellum	Astrogliosis and spongiosis, predominantly in the brainstem and cerebellum
Histopathological signs in the kidney	No alterations detected	Glomerular hypertrophy	No alterations detected

4-HBA treatment = *Coq2^A252V^* + 4-HBA; CoQ_10_ treatment = *Coq2^A252V^* + CoQ_10_; and 4-HBA withdrawal after 90 days of treatment = *Coq2^A252V^* − 4-HBA. 4-HBA = 4-hydroxybenzoic acid.

### Patient report (pre-4-HBA treatment)

We subsequently identified a patient potentially amenable to treatment with 4-HBA. The patient is the first child of healthy, non-consanguineous parents of German descent. Pregnancy and birth were uneventful. During the first year of life, developmental delay and muscular hypotonia became apparent. He achieved unsupported sitting at 12 months and began crawling at 17 months. Speech development was also delayed. At the age of 2 years and 6 months, he was able to articulate approximately 15 single words. In addition to developmental delays, recurrent vomiting and failure to thrive were noted. Laboratory investigations revealed persistent lactic acidosis. At the age of 2 years and 8 months, the child developed generalized oedema, and nephrotic syndrome was diagnosed. Treatment with steroids failed to elicit any improvement.

**Figure 4:**
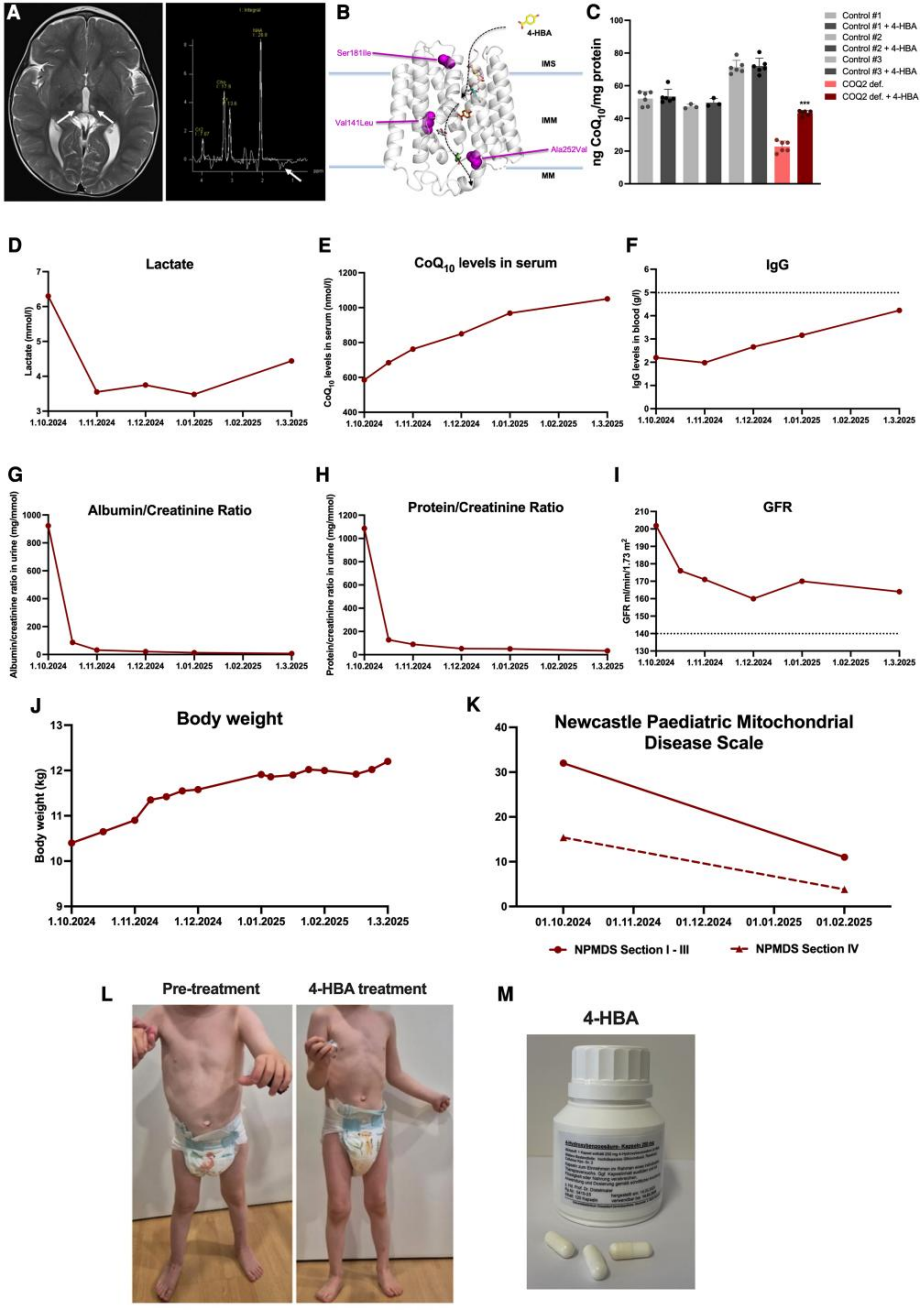
**Effects of 4-HBA treatment in a child with primary CoQ deficiency due to compound heterozygous variants in *COQ2*.** (**A**) Brain MRI image (T2-weighted, axial view) and MR spectroscopy at the age of 3 years 3 months, showing symmetrical lesions in the thalami (arrows; Leigh-syndrome pattern) as well as a small lactate peak detected in the white matter (arrow). (**B**) *In silico* model of the COQ2 enzyme depicting the patient variants and the variant of the mouse model (in purple). The putative 4-HBA transportation pathway is indicated (arrow with dotted line). IMS = intermembrane space; IMM = inner mitochondrial membrane; MM = mitochondrial matrix. (**C**) Quantification of CoQ_10_ levels in control and COQ2-deficient fibroblast cell lines measured by UPLC-MS/MS. Bar graphs depict untreated (light shaded bars) and treated (dark shaded bars) conditions. 4-HBA was used at a concentration of 100 µmol for 14 days. Comparison of the treated patient-derived cells (light red shaded bar, COQ2 def.) compared to the 4-HBA treated condition (dark red shaded bar, COQ2 def. 4-HBA). ***P < 0.001. Statistical significance was assessed using Student’s *t*-test. Experimental data were obtained in three independent experiments. *n* = 3–6. (**D**) Lactate levels measured in serum during the course of 4-HBA treatment. The graph shows monthly measurements from 1 Oct 2024 to 1 Mar 2025. (**E**) CoQ_10_ levels measured in serum during the course of 4-HBA treatment. The graph shows monthly measurements from 1 Oct 2024 to 1 Mar 2025. (**F**) Immunoglobulin G (IgG) levels in blood during the course of treatment (dotted line = lower limit of normal range). (**G**) Albumin/creatinine ratio and (**H**) protein/creatinine ratio in urine during the course of treatment. (**I**) Glomerular filtration rate (GFR) as an indicator of renal hyperfiltration during the course of treatment (dotted line = upper limit of normal range). (**J**) Record of the body weight of the patient during the treatment period. (**K**) NPMDS (Newcastle Paediatric Mitochondrial Disease Scale) scores at the start and after 5 months of treatment. (**I**) Clinical images of the affected child. *Left*: Pretreatment condition at the initiation of 4-HBA therapy (age 3 years 3 months). *Right*: Condition after 5 months of 4-HBA treatment (age 3 years 6 months). Improved muscle strength (unsupported standing) and muscle relief are visible (see also Supplementary Videos 5 and 6). (**M**) Image of the encapsulation of 4-HBA at specific doses of 250 mg for clinical use. Excipients are indicated. 4-HBA = 4-hydroxybenzoic acid; def. = deficiency; UPLC-MS-MS = ultra-high performance tandem mass spectrometry.

At 3 years of age, a metabolic disorder was suspected, and exome sequencing was performed. Genetic analysis revealed compound-heterozygous variants in the *COQ2* gene [NM_001358921.2:c.421G>C, p.(Val141Leu),^24^ old nomenclature: NM_015697.9: c.571G>C, p.(Val191Leu), maternal; NM_001358921.2:c.542G>T, p.(Ser181Ile), old nomenclature: NM_015697.9:c.692G>T, p.(Ser231Ile), paternal]. The c.421G>C variant introduces an amino acid change in a highly conserved region across species and is predicted to affect protein function according to bioinformatics tools [REVEL (Rare Exome Variant Ensemble Learner) score: 0.852, CADD (Combined Annotation Dependent Depletion) score: 33]. The affected residue lies within the prenyltransferase domain (amino acids 44–371, Pfam: PF01040), which is essential for enzymatic activity of COQ2. The variant is absent in the general population (gnomAD; minor allele frequency 0%). Together with the clinical phenotype of the patient and biochemical evidence of CoQ deficiency, the variant was considered as likely pathogenic. The variant c.542G>T also leads to an amino acid change and has already been described to be pathogenic in the literature^25^ (REVEL score: 0.925, CADD score: 37). None of the *COQ2* variants were detected in the unaffected younger brother. In addition, a variant of unknown significance was identified in *ATP2B3* [NM_001001344.3:c.2419G>A, p.(Val807Met)], hemizygous, maternally inherited, which was not considered to be disease-relevant. Brain MRI at the age of 3 years 3 months revealed symmetrical thalamic lesions consistent with a Leigh-like pattern, along with a moderate lactate peak on MR spectroscopy (Fig. 4A).

Based on the clinical and molecular findings, a diagnosis of primary CoQ deficiency-1 (COQ10D1; OMIM #607426), caused by pathogenic variants in *COQ2*, was established. Thus, oral supplementation with CoQ_10_ was initiated at a dose of 30 mg/kg body weight/day. Moreover, a medication with ramipril was started to improve proteinuria. Over the course of 3 months, the parents reported mild improvements, including increased activity, fewer episodes of vomiting and reduced apathy, although no major clinical changes were observed. Renal function showed slight improvement, reflected by a decrease in glomerular filtration rate (GFR), suggestive of improved hyperfiltration.

### *In silico* modelling of *COQ2* variants and biochemical analysis of 4-HBA effects in patient-derived skin fibroblasts

Based on our previous structural analysis of *COQ2* variants using an *in silico* model of the COQ2 enzyme, we investigated the spatial location of the patient’s variants (Fig. 4B).^13^ The variants identified in this patient—p.Val141Leu and p.Ser181Ile—are located in close proximity to the putative substrate transport tunnel.^13^ Bioinformatic predictions suggested that the p.Val141Leu substitution may impair substrate binding (Fig. 4B). The Val141Leu variant is localized, similar to the Ala252Val variant, in close proximity to the active site of the protein. This likely has a direct impact on the biochemical reaction, for example the velocity which might be reduced due to an altered binding of the substrate. The p.Ser181Ile variant may interfere with substrate entry into the tunnel leading to reduced uptake of the substrate and thereby lower availability at the active site.

To assess the functional impact of these variants and the potential therapeutic effect of 4-HBA, we obtained a skin biopsy from the patient. Cultured fibroblasts were treated with or without 4-HBA, and CoQ_10_ levels were quantified in both patient-derived and control fibroblasts. CoQ_10_ deficiency was confirmed in the patient’s fibroblasts (Fig. 4C). Treatment with 4-HBA led to a marked increase in endogenous CoQ_10_ biosynthesis in patient fibroblasts (Fig. 4C), replicating the findings previously reported.^4^

### Clinical application of 4-HBA therapy

The proposed treatment attempt with 4-HBA was discussed with the family, who provided written informed consent to initiate the individual therapeutic trial. Prior to initiation, comprehensive baseline laboratory assessments were performed, revealing lactic acidosis (levels between 5 and 6 mmol/l, norm <1.60; base excess around −10 mmol/l, norm between −2 and +2 mmol/l; Fig. 4D), low serum CoQ_10_ levels (Fig. 4E), low blood IgG levels (Fig. 4F), marked albuminuria and proteinuria (albumin/creatinine ratio in urine 922.8 mg/mmol, norm <2.3, Fig. 4G; protein/creatinine ratio in urine 1086.1 mg/mmol, norm <20, Fig. 4H) and high GFR (Fig. 4I). Additional evaluations (e.g. abdominal ultrasound or echocardiography) were unremarkable. Body weight was low for his age (Fig. 4J). The Newcastle Paediatric Mitochondrial Disease Scale (NPMDS) was also completed (Fig. 4K). The patient underwent assessments by the physiotherapy and speech therapy teams. Clinical photographs (Fig. 4L, left) and video documentation (Supplementary Video 5) were obtained.

Oral CoQ_10_ supplementation was discontinued prior to initiating 4-HBA therapy. Treatment with 4-HBA was started at the age of 3 years and 3 months and initiated orally at a dose of 3 × 250 mg/day (75 mg/kg body weight/day) and was increased after 1 week to 3 × 500 mg/day (150 mg/kg body weight/day). Encapsulation of 4-HBA at specific dosages was performed by the hospital pharmacy (Fig. 4M). To mitigate the acidity of the compound, 4-HBA was dissolved in milk. This approach proved well tolerated without any clinical issues.

Throughout the treatment period, laboratory parameters were monitored closely. Lactate levels drastically decreased 1 month after the treatment (lactate levels around 3 mmol/l, base excess around −4 mmol/l; Fig. 4D). Serum CoQ_10_ levels rose progressively, reaching levels above 1 µmol/l (Fig. 4E). Hypogammaglobulinaemia showed a trend towards normal values (Fig. 4F). A rapid and pronounced improvement in renal parameters was observed, i.e. albuminuria and proteinuria markedly decreased within 3 weeks (Fig. 4G and H), allowing for discontinuation of ramipril treatment. Concurrently, renal hyperfiltration improved as evidenced by a tendency toward normalization of GFR (Fig. 4I).

No adverse effects of 4-HBA treatment were observed during 6 months of follow-up. Routine laboratory investigations remained within normal ranges (including haematology, electrolytes, liver function tests and coagulation profiles).

Clinically, both the family and therapists noted striking improvements in muscle strengths, mobility, exercise tolerance, and fine motor function. Feeding behaviour improved substantially, with cessation of recurrent vomiting and a weight gain of 2 kg over 4 months (Fig. 4J). The parents also reported enhanced hair and nail growth, likely reflecting improved nutritional status. These improvements were mirrored by a significant reduction in the NPMDS score (Fig. 4K). The patient, who had previously been unable to walk unaided, was able to walk independently for extended distances after 4 months of 4-HBA treatment (Fig. 4L, right and Supplementary Video 6). Cognitive and developmental progress was also evident. The patient expanded his vocabulary to 70–80 single words and began using two-word sentences. Alertness, concentration, and social interaction improved noticeably. Whereas he had previously exhibited marked anxiety and limited age-appropriate play, he now engaged actively and responded well to therapeutic interventions.

Currently, 4-HBA treatment is ongoing for 9 months and is planned to be sustained in the future.

## Discussion

Despite significant advances in our molecular understanding of mitochondrial diseases, therapeutic options remain limited. This is particularly evident in primary CoQ deficiency syndrome, where high-dose oral CoQ_10_ supplementation, the current standard of care, fails to halt disease progression, especially in cases with CNS involvement.^7^ The limited efficacy of CoQ_10_ is largely attributed to its suboptimal pharmacokinetic properties, including poor bioavailability and restricted tissue penetration.^7,8^ To overcome these limitations, we focused on enhancing endogenous CoQ biosynthesis by targeting COQ2, a key enzyme in the early steps of the biosynthetic pathway. COQ2 activity depends on the availability of its substrates, 4-HBA and the polyisoprenoid tail, with 4-HBA representing a critical rate-limiting factor. Accordingly, we previously demonstrated that pathogenic *COQ2* variants impair substrate transport within the enzyme, and that increasing 4-HBA concentrations could restore enzymatic function in patient-derived fibroblasts and *in vivo* models.^13,14^ Following up on these initial results, we now show that lifelong 4-HBA supplementation in *Coq2^A252V^* mice is necessary to maintain metabolic and neurological homeostasis. Treatment withdrawal in adult animals led to rapid onset of mitochondrial encephalopathy, characterized by gliosis and spongiosis, consistent findings reported in other models of mitochondrial diseases.^20,21,26,27^ This underscores the sustained requirement for functional CoQ biosynthesis across developmental stages. Notably, this phenotype mirrors the neurodegeneration seen in patients with multiple system atrophy linked to *COQ2* variants in the Asian population,^6,28^ suggesting a possible therapeutic role for 4-HBA in this adult-onset phenotype.^29^

Importantly, our results also demonstrate the superiority of 4-HBA over conventional CoQ_10_ treatment. While CoQ_10_ transiently improved cardiac development in *Coq2^A252V^* embryos, it failed to prevent fatal neurodegeneration with pronounced gliosis in the brainstem and cerebellum in born *Coq2^A252V^* mice. Interestingly, this clinical course closely resembles what is observed in patients with primary CoQ deficiency, in whom a slight initial improvement may be observed following CoQ_10_ supplementation, although without achieving full recovery or halting disease progression.^7^ Also, these findings are consistent with previous reports showing that exogenous CoQ_10_ primarily accumulates in the liver and poorly penetrates the brain.^8,30,31^ In contrast, 4-HBA restored CoQ biosynthesis systemically, improved mitochondrial respiratory chain activity and sustained survival, neurodevelopment and brain homeostasis, suggesting that 4-HBA, as other related phenolic compounds,^32^ crosses the blood–brain barrier and stimulates CoQ biosynthesis in the CNS, although the specific mechanisms by which 4-HBA crosses the blood–brain barrier need to be investigated. Remarkably, functional recovery was achieved even though brain CoQ levels did not reach those observed in wild-type animals. This can be explained by two factors: (i) 4-HBA likely stimulates CoQ biosynthesis specifically within mitochondria, where CoQ exerts its critical metabolic roles^1^; and (ii) under physiological conditions, cells may synthesize CoQ in excess of their minimal functional requirements, as suggested by the absence of a fatal phenotype in the *Coq9^Q95X^* mouse model, which retains approximately 40% of normal CoQ levels.^33^

In line with our findings, a study conducted independently and in parallel to our work was recently published,^34^ indicating that 4-HBA supplementation may also be beneficial in genetic disorders beyond COQ2.^34^ Early-life administration of 4-HBA in *Hpdl*^−/−^ mice, as well as in a single individual with HPDL deficiency, was sufficient to partially restore CoQ levels, significantly extend survival in the murine model, and improve clinical outcomes in the patient.^34^ These results suggest that 4-HBA may also be effective in defects impacting its own biosynthetic pathway. However, our data indicate that in COQ2 deficiency, continuous, lifelong supplementation is required to sustain therapeutic benefit, whereas in HPDL deficiency, 4-HBA appears to be necessary only during early developmental stages.^34^ These contrasting requirements underscore the importance of a personalized treatment approach, guided at minimum by the specific gene affected. On the contrary, 4-HBA is unlikely to be effective in defects affecting downstream steps of the CoQ biosynthetic pathway, as it acts upstream and cannot bypass enzymatic blocks occurring later in the process. In fact, in some contexts, such as the *Coq9^R239X^* mouse model, 4-HBA supplementation may exacerbate the accumulation of DMQ, a potentially toxic intermediate.^16^ Therefore, 4-HBA treatment should be restricted to cases with confirmed *in vitro* responsiveness, reinforcing the need for gene-specific, precision therapies. Interestingly, other 4-HBA analogues have demonstrated good therapeutic efficacy in patients-derived fibroblasts and mouse models harbouring specific defects in the later steps of the CoQ biosynthetic pathway.^16,17,33,35-39^

Building on the promising results obtained from patient-derived fibroblasts and the *Coq2^A252V^* mouse model, we then evaluated the translational potential of 4-HBA treatment to humans. Despite encouraging preclinical evidence, it remained uncertain whether the patient would tolerate 4-HBA, especially given its lack of prior clinical use. Moreover, unlike the mouse model, a patient already exhibits clinical symptoms, raising concerns about the reversibility of the disease. Remarkably, 4-HBA treatment fully restored renal function and led to rapid neurological improvement in the patient described here, highlighting its strong therapeutic potential. Clinically, the speed of response was reminiscent of that seen in transporter-related disorders, such as thiamine transporter deficiency.^40^ This rapid onset of action further supports the hypothesis that COQ2 may possess a transporter-like function for 4-HBA. Mechanistically, this observation may also reflect an increase in 4-HBA concentration beyond the *K*_m_ of COQ2, thereby enhancing its enzymatic activity. Of note, the Val141Leu variant is localized, similar to the Ala252Val variant, in close proximity to the active site of the protein. Therefore, it might have a direct impact on the biochemical reaction. This could constitute a problem regarding the efficiency of 4-HBA treatment. However, the variant Ala252Val was investigated in a homozygous state in patient fibroblasts^36^ and in the current mouse model. Despite its location close to the active site, the biochemical problem was clearly amendable by 4-HBA treatment in both model systems. This suggests that 4-HBA transport or binding might be impaired. In a previous study on the *in vitro* effects of 4-HBA in COQ2-deficient fibroblast lines, we developed an *in silico* model of COQ2 protein structure and investigated the spatial distribution of known pathogenic *COQ2* variants in relation to the predicted 4-HBA transport tunnel. We observed that the variants that are associated with severe, early-onset phenotypes tend to cluster near the transport tunnel, likely interfering with 4-HBA transport or binding to the catalytic site. In contrast, variants linked to later-onset phenotypes were located further away from the tunnel, possibly altering overall protein conformation and thereby indirectly impairing 4-HBA access. Importantly, none of the analysed variants appeared to fully disrupt the catalytic site of the COQ2 enzyme, suggesting that such variants may be incompatible with life. Based on these observations, we propose that impaired 4-HBA transport and/or binding at the active site are key pathomechanisms in human COQ2 deficiency. Consequently, the majority of the known *COQ2* variants should be amendable to therapeutic rescue by 4-HBA treatment. Nevertheless, the possibility that certain variants are unresponsive to 4-HBA cannot be excluded. Of note, this concept of substrate enhancement treatment has yielded promising therapeutic outcomes in other mitochondrial diseases as well, such as the supplementation of pyrimidine nucleosides in both mouse model and patients with pathogenic variants in thymidine kinase 2.^41,42^ This further supports the notion of a promising perspective for 4-HBA treatment in COQ2 deficiency.

Despite its classification as a hazardous compound, due to its potential to cause skin and eye irritation, our clinical experience with oral administration revealed no apparent adverse effects. Handling of the compound by the patient’s caregivers was reported to be straightforward. Nevertheless, families must be appropriately counselled regarding the risks associated with 4-HBA powder. Clinical and laboratory follow-up over a 9-month period revealed no signs of toxicity, consistent with findings from animal studies and previous short-term human applications. These findings, together with its GRAS status and natural occurrence, support its suitability for further clinical development. Nevertheless, long-term safety, including carcinogenic potential, remains to be systematically evaluated. Manufacturing under Good Manufacturing Practice (GMP) conditions and formal clinical trials will be essential next steps toward broader therapeutic implementation.

In conclusion, our study provides robust preclinical and clinical evidence supporting the safety and efficacy of 4-HBA supplementation as a novel therapeutic strategy to enhance endogenous CoQ biosynthesis in individuals harboring pathogenic *COQ2* variants. Further investigations will be critical to assess the therapeutic applicability of 4-HBA in other forms of primary CoQ deficiency caused by upstream defects in the biosynthetic pathway, and to pave the way for regulatory approval and clinical translation.

## Supplementary Material

awaf334_Supplementary_Data

## Data Availability

The data that support the findings of this study are available upon reasonable request.
