# Supplementary material for: Preclinical and first-in-human evidence of 4-hydroxybenzoic acid for mitochondrial COQ2 deficiency
Source: Brain. 2025 Sep 10;149(5):1784–97. doi: 10.1093/brain/awaf334 (PMC13140534; doi:10.1093/brain/awaf334)
Supplement: awaf334_Supplementary_Data [file awaf334_supplementary_data.zip › brain-2025-01179-File007.pdf]

Supplementary material

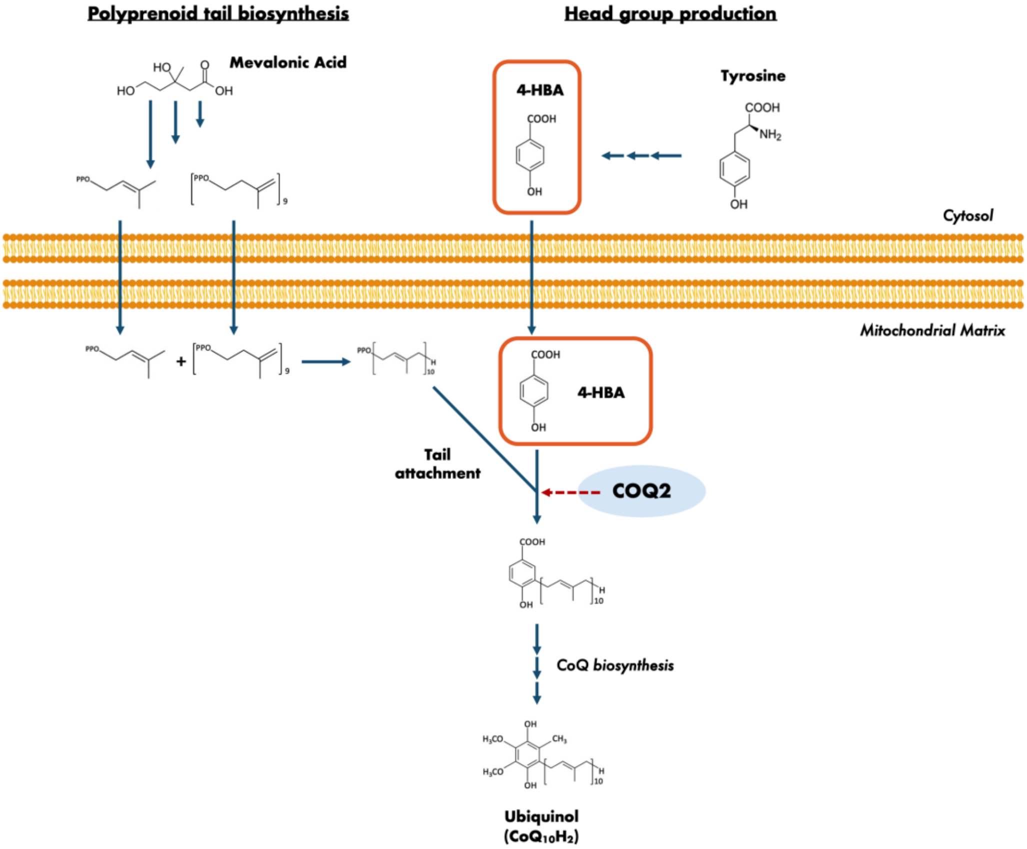

Supplementary Figure 1. Summary of the CoQ biosynthetic pathway, remarking the reaction catalyzed by COQ2.

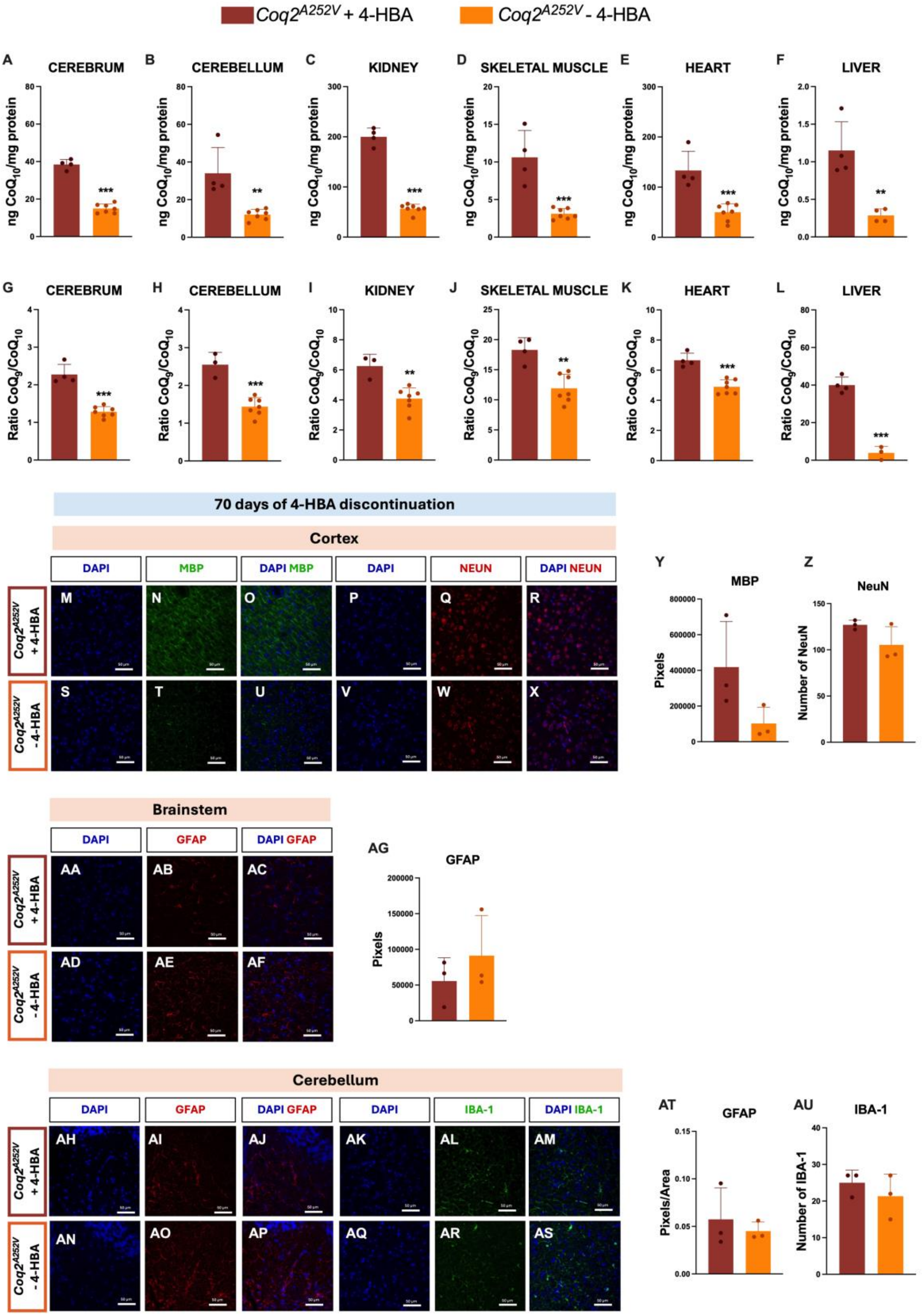
Coq2A252V + 4-HBA
Coq2A252V - 4-HBA
A CEREBRUM
B CEREBELLUM
C KIDNEY
D SKELETAL MUSCLE
E HEART
F LIVER
ng CoQ10/mg protein
G CEREBRUM
H CEREBELLUM
I KIDNEY
J SKELETAL MUSCLE
K HEART
L LIVER
Ratio CoQ9/CoQ10
70 days of 4-HBA discontinuation
Cortex
DAPI
MBP
DAPI MBP
DAPI
NEUN
DAPI NEUN
Coq2A252V + 4-HBA
Coq2A252V - 4-HBA
Y MBP
Pixels
Z NeuN
Number of NeuN
Brainstem
DAPI
GFAP
DAPI GFAP
AG GFAP
Pixels
Cerebellum
DAPI
GFAP
DAPI GFAP
DAPI
IBA-1
DAPI IBA-1
AT GFAP
Pixels/Area
AU IBA-1
Number of IBA-1

Supplementary Figure 2: Assessment of CoQ metabolism and neuroinflammation progression in *Coq2^{A252V}* mice without 4-HBA after 70 days without treatment.

(**A-F**) CoQ_{10} levels in cerebrum (A), cerebellum (B), kidney (C), skeletal muscle (D), heart (E) and liver (F) at 70 days with and without 4-HBA treatment (n = 4 - 7 in each experimental group).

(**G-L**) CoQ_9/CoQ_{10} ratio in cerebrum (G), cerebellum (H), kidney (I), skeletal muscle (J), heart (K) and liver (L) at 70 days with and without 4-HBA treatment (n = 3 - 7 in each experimental group).

(**M-R**) MBP stain (M-O) and NeuN stain (P-R) in the cortex of *Coq2^{A252V}* mice treated with 4-HBA at 70 days post-withdrawal (n = 3 in each experimental group).

(**S-X**) MBP stain (S-U) and NeuN stain (V-X) in the cortex of *Coq2^{A252V}* after 4-HBA discontinuation at 70 days post-withdrawal (n = 3 in each experimental group).

(**Y**) Quantification of MBP in cortex of *Coq2^{A252V}* mice treated with 4-HBA and after 4-HBA discontinuation at 70 days post-withdrawal (n = 3 in each experimental group).

(**Z**) Quantification of NeuN in cortex of *Coq2^{A252V}* mice treated with 4-HBA and after 4-HBA discontinuation at 70 days post-withdrawal (n = 3 in each experimental group).

(**AA-AF**) GFPA stain in the brainstem of *Coq2^{A252V}* mice treated with 4-HBA (Y-AA) and after 4-HBA discontinuation (AB-AD) at 70 days post-withdrawal

(**AG**) Quantification of GFAP in brainstem of *Coq2^{A252V}* mice treated with 4-HBA and after 4-HBA discontinuation at 70 days post-withdrawal (n = 3 in each experimental group).

(**AH-AM**) GFAP stain (AH-AJ) and IBA-1 stain (AK-AM) in the cerebellum of *Coq2^{A252V}* mice treated with 4-HBA at 70 days post-withdrawal withdrawal (n = 3 in each experimental group).

(**AN-AS**) GFAP stain (AN-AP) and IBA-1 stain (AQ-AS) in the cerebellum of *Coq2^{A252V}* mice after 4-HBA discontinuation at 70 days post-withdrawal withdrawal (n = 3 in each experimental group).

(**AT**) Quantification of GFAP in cerebellum of *Coq2^{A252V}* mice treated with 4-HBA and after 4-HBA discontinuation at 70 days post-withdrawal (n = 3 in each experimental group).

(**AU**) Quantification of IBA-1 in cerebellum of *Coq2^{A252V}* mice treated with 4-HBA and after 4-HBA discontinuation at 70 days post-withdrawal (n = 3 in each experimental group).

Scale bars: 50 μm (M-X, AA-AF, AH-AS)

Coq2^{A252V} mice were treated with 4-HBA for 90 days, given the 4-HBA in the chow at a concentration of 0,33% (w/w). At that point, the treatment was discontinued and replaced with standard animal facility chow in the *Coq2*^{A252V} without 4-HBA group. Data are represented as mean ± SD. **p < 0.01 vs. *Coq2*^{A252V} treated with 4-HBA; ***p < 0.001 vs. *Coq2*^{A252V} treated with 4-HBA.

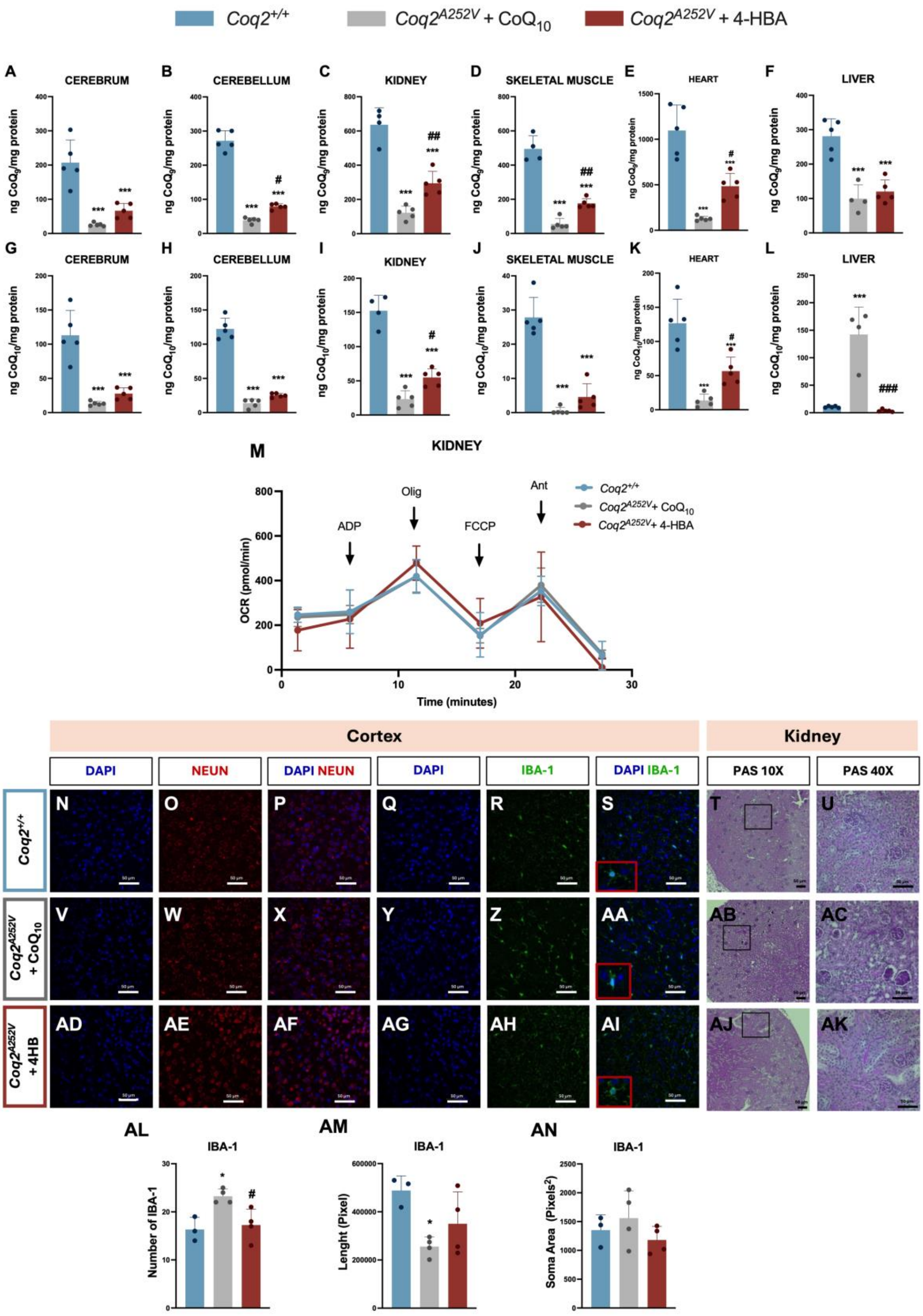

Supplementary Figure 3. Evaluation of mitochondrial bioenergetic, neuroinflammation and kidney morphology in the *Coq2^{A252V}* model with CoQ$_{10}$ and 4-HBA treatments

(**A-F**) CoQ_9 levels in cerebrum (A), cerebellum (B), kidney (C), skeletal muscle (D), heart (E) and liver (F) (n = 4 - 5 in each experimental group).
(**G-L**) CoQ_{10} levels in cerebrum (G), cerebellum (H), kidney (I), skeletal muscle (J), heart (K) and liver (L) (n = 4 - 5 in each experimental group).
(**M**) Mitochondrial oxygen consumption rate (represented as State 3o, in the presence of ADP and substrates) in kidney (n = 3 in each experimental group).
(**N-S**) NeuN stain (N-P) and IBA-1 stain (Q-S) in the cortex of $Coq2^{+/+}$ mice at 21 days of age (n = 3 - 5 in each experimental group).
(**T-U**) PAS stain in the kidney of $Coq2^{+/+}$ mice at 21 days of age (n = 3 - 5 in each experimental group).
(**V-AA**) NeuN stain (V-X) and IBA-1 stain (Y-AA) in the cortex of $Coq2^{A252V}$ treated with CoQ_{10} mice at 21 days of age (n = 3 - 5 in each experimental group).
(**AB-AC**) PAS stain in the kidney of $Coq2^{A252V}$ treated with CoQ_{10} mice at 21 days of age (n = 3 - 5 in each experimental group).
(**AD-AI**) NeuN stain (AD-AF) and IBA-1 stain (AG-AI) in the cortex of $Coq2^{A252V}$ treated with 4-HBA mice at 21 days of age (n = 3 - 5 in each experimental group).
(**AJ-AK**) PAS stain in the kidney of $Coq2^{A252V}$ treated with 4-HBA mice at 21 days of age (n = 3 - 5 in each experimental group).
(**AL**) Quantification of IBA-1 in cortex of $Coq2^{+/+}$, $Coq2^{A252V}$ treated with CoQ_{10} and $Coq2^{A252V}$ treated with 4-HBA mice at 21 days of age (n = 3 - 5 in each experimental group).
(**AM**) Quantification of the length of IBA-1 in pixels in cortex of $Coq2^{+/+}$, $Coq2^{A252V}$ treated with CoQ_{10} and $Coq2^{A252V}$ treated with 4-HBA mice at 21 days of age (n = 3 - 5 in each experimental group).
(**AN**) Quantification of the soma area of IBA-1 in pixels in cortex of $Coq2^{+/+}$, $Coq2^{A252V}$ treated with CoQ_{10} and $Coq2^{A252V}$ treated with 4-HBA mice at 21 days of age (n = 3 - 5 in each experimental group).
Scale bars: 50 µm (N-AK).
CoQ_{10} and 4-HBA was given to the mice in the chow at a concentration of 0.33% (w/w). Data are represented as mean ± SD. *$p < 0.05$ vs. $Coq2^{+/+}$; **$p < 0.01$ vs. $Coq2^{+/+}$; ***$p < 0.001$ vs. $Coq2^{+/+}$; #$p < 0.05$ vs. $Coq2^{A252V}$ treated with CoQ_{10}; ##$p < 0.01$ vs. $Coq2^{A252V}$ treated with CoQ_{10}; and ###$p < 0.001$ vs. $Coq2^{A252V}$ treated with CoQ_{10}.

Multimedia Files

Supplementary video 1. *Coq2^{A252V}* mice 210 after 4-HBA therapy days discontinuation.

Supplementary video 2. *Coq2$^{+/+}$* mice, *Coq2^{A252V}* treated with CoQ_{10} mice and *Coq2^{A252V}* treated with 4-HBA mice at 21 days of age.

Supplementary video 3. Pole test performance of *Coq2^{A252V}* treated with CoQ_{10} mice and *Coq2^{A252V}* treated with 4-HBA mice at 21 days of age.

Supplementary video 4. Spontaneous behavior of *Coq2^{A252V}* treated with CoQ_{10} mice at 21 days of age

Supplementary video 5. Mobility of the *COQ2* patient before the start of 4-HBA treatment.

Supplementary video 6. Mobility of the *COQ2* patient after 4 months of 4-HBA treatment.
